# Eligibility and Enrollment in the Special Supplemental Nutrition Program for Women, Infants, and Children (WIC) — 27 States and New York City, 2007–2008

**Published:** 2013-03-15

**Authors:** Kristen S. Marchi, Paula A. Braveman, Katie Martin, Michael Curtis, Tonya Stancil, Leslie Harrison

**Affiliations:** Dept of Family and Community Medicine, Univ of California, San Francisco; Maternal, Child and Adolescent Health Program, California Dept of Public Health; Div of Reproductive Health, CDC

The national Special Supplemental Nutrition Program for Women, Infants, and Children (WIC) provides nutrition education, growth monitoring, breastfeeding promotion and support, and food to low-income pregnant or postpartum women, infants, and children aged <5 years. Several studies have linked WIC services with improved maternal and infant health outcomes (1–3). Most population-based studies have lacked information needed to identify eligible women who are not receiving WIC services and might be at risk for poor health outcomes. This report uses multistate, population-based 2007–2008 survey data from CDC’s Pregnancy Risk Assessment Monitoring System (PRAMS) and California’s Maternal and Infant Health Assessment (MIHA) to estimate how many women were eligible but not enrolled in WIC during pregnancy and to describe their characteristics and their prevalence of markers of risk for poor maternal or infant health outcomes (4–6). Approximately 17% of all women surveyed were eligible but not enrolled in WIC during pregnancy. The proportion of women eligible for WIC and WIC participation rates varied by state. WIC participants had higher prevalences of markers of risk for poor maternal or infant health outcomes than eligible nonparticipants, but both groups had higher prevalences of risk markers than ineligible women, suggesting that many eligible women and their children might benefit from WIC services. The results of this analysis can help identify the scope of WIC outreach needed to include more eligible nonparticipants in WIC and whom to target.

This study’s sample included 71,267 women who participated in CDC’s PRAMS survey in 26 states and New York City, and 6,435 women who participated in California’s MIHA during 2007 or 2008 (Table 1). The two separate surveillance systems, PRAMS and MIHA, conduct annual, population-based mail surveys of women with recent live births sampled from birth certificates, with telephone follow-up of nonrespondents. The surveys used in this study include many similar questions, use similar methods (7), and have response rates of at least 65%.

Women reporting WIC participation at any time during their most recent pregnancies were classified as WIC participants. WIC eligibility requires a household income ≤185% of the federal poverty level (FPL)* or participation in another program (e.g., Medicaid) with similar income criteria. WIC nonparticipants were considered eligible if they reported incomes ≤185% FPL in the survey or if the birth certificate indicated Medicaid payment for prenatal care or delivery. Nonparticipants in WIC or Medicaid with incomes >185% FPL were considered ineligible. Women with missing information on WIC enrollment, insurance, or income (n = 1,653) were excluded, yielding a final sample of 76,049 women, which is representative of a total of 4,023,136 live births to resident women in these states, approximately half of all births in the United States during 2007–2008.

WIC participants and eligible nonparticipants as a proportion of all women delivering a live infant and as a percentage of all eligible women delivering a live birth were examined overall, then in each state. In the overall sample, WIC participants, eligible nonparticipants, and ineligible women were then compared on social characteristics important for targeting programs (e.g., race/ethnicity and language) or for assessing potential need for WIC services, as indicated by well-documented markers of risk for adverse maternal or infant health outcomes (4–6) (Table 2). Markers of risk included 1) having less than a high school education or being aged <18 years, 2) having delivered four or more live infants, 3) being unmarried at time of delivery, 4) being poor (income ≤100% FPL), 5) having Medicaid or no health-care coverage before pregnancy, 6) having no prenatal care in the first or second trimester, 7) having an unintended pregnancy, 8) being either underweight or obese before pregnancy, 9) smoking before pregnancy, and 10) having a history of delivering an infant preterm (before 37 weeks completed gestation) or of low birth weight (<2,500 g) (4,5). Finally, the percentage of women in each group with one, two, three, or four or more of the risk markers was examined. Prenatal health-care coverage was not included in the sum of the risk markers because it was used to define the WIC groups (Table 2). All estimated counts, percentages, and 95% confidence intervals were weighted to represent all live births in the participating states using statistical survey procedures that account for complex sample design.

Among all women surveyed, 46% were WIC participants, approximately 17% were classified as eligible nonparticipants (Table 1), and 37% were classified as ineligible (Table 2). Variation by state was evident in the percentage of all women delivering a live infant who were enrolled in WIC during pregnancy, from a low of 28% in Utah to a high of 57% in Oklahoma, and in the percentage of all women classified as WIC-eligible but who were not enrolled, from a low of 11% in Rhode Island to a high of 31% in Utah (Table 1). The proportion of all eligible women enrolled in WIC was approximately 74% overall, varying from a low of 48% in Utah to a high of 83% in California (Table 1).

Nearly one fifth (19%) of WIC participants were non-Hispanic blacks and 39% were Hispanics, compared with 14% and 21% of eligible nonparticipants and 5% and 7% of ineligible women, respectively (Table 2). Conversely, WIC participants included a lower proportion of non-Hispanic white women (35%) than was found among eligible nonparticipants (57%), or among ineligible women (76%). Approximately 25% of WIC participants completed the survey in Spanish, compared with 12% of eligible nonparticipants and <2% of ineligible women.

Overall, the risk characteristics of WIC participants and eligible nonparticipants differed from those of ineligible women (Table 2). WIC participants generally appeared to be at greater social and economic disadvantage, as measured by indicators of risk for delivering a preterm or low birth weight infant, than were eligible nonparticipants. WIC participants and eligible nonparticipants were more disadvantaged than ineligible women, as reflected by their low incomes and the proportion of women who had <12 years of education, were aged <18 years, had four or more live births, were unmarried, had Medicaid or no health-care coverage before pregnancy, or initiated prenatal care in the third trimester or not at all (Table 2). WIC participants and eligible nonparticipants also had higher prevalences of other health risks than ineligible women, as reflected, for example, by prepregnancy obesity, smoking before pregnancy, and a previous low birth weight or preterm birth.

WIC participants and eligible nonparticipants appeared to be at risk for poor maternal or infant outcomes, based on markers of risk (Table 2). Approximately 91% of eligible nonparticipants had at least one risk marker, and 75% reported at least two markers, compared with 97% and 90% of WIC participants, respectively. Among eligible nonparticipants, 36% reported four or more risk markers, compared with 54% of WIC participants. WIC-ineligible women reported markedly fewer risk characteristics than women in the other two groups.

## Editorial Note

The results of this analysis indicate that, although WIC covered most eligible women overall and in many states during 2007–2008, an estimated 662,800 eligible women were not enrolled in WIC in the 27 states examined. The proportion of eligible women who were enrolled in WIC varied widely by state. Overall, the findings indicate that WIC is enrolling high-risk women and reveal that most eligible nonparticipants also have social and economic characteristics that repeatedly have been linked to adverse maternal or infant health outcomes. In addition, WIC participants and eligible nonparticipants have higher rates of other health risks, such as prepregnancy obesity and previous poor birth outcomes, than ineligible women. Three quarters of eligible nonparticipants had two or more markers of risk; more than one third had four or more. Although WIC’s services cannot address all relevant risks, promoting and supporting more adequate nutrition might improve some health outcomes among vulnerable women and their children during the critical periods of pregnancy and infancy, with potentially lifelong benefits (8–10). Referrals by WIC to outside services, such as prenatal care and smoking cessation programs, also could benefit women, infants and children in the long run.

The findings in this report are subject to at least four limitations. First, the study relied on unverified self-reports of income and WIC participation. Second, PRAMS and MIHA measure average income over 1 year, which might underestimate WIC eligibility. Third, health-care coverage can change during pregnancy, affecting the ability to determine eligibility for WIC. Finally, although survey response rates were at least 65%, differences might exist between the respondents and nonrespondents. This concern was mitigated through nonresponse weighting of the survey data, by which differing weights were assigned to demographic groups with significantly different response rates.

What is already known on this topic?

The Special Supplemental Nutrition Program for Women, Infants, and Children (WIC) provides nutrition education, growth monitoring, breastfeeding promotion and support, and food to low-income pregnant or postpartum women, infants, and children aged <5 years. Several studies have linked WIC services with improved maternal and infant health.

What is added by this report?

Among women from 27 states and New York City who participated in a survey of mothers who had recently delivered a live infant during 2007–2008, 46% were WIC participants and approximately 17% were classified as eligible nonparticipants. WIC participants generally were at greater social and economic disadvantage than were eligible nonparticipants, as measured by indicators of risk for delivering a preterm or low birth weight infant, but both groups were more disadvantaged than ineligible women.

What are the implications for public health practice?

Efforts to expand outreach to eligible non-WIC participants could improve maternal and infant health outcomes among low-income pregnant or postpartum women, infants, and children aged <5 years. The results of this analysis can help identify the scope of WIC outreach needed and whom to target.

The large size of the WIC-eligible population reflects levels of poverty (<100% FPL) and near-poverty (101%–185% FPL) around the time of pregnancy, confirming previous findings that many women giving birth in the United States are poor or near-poor (7). Given current economic conditions, it is possible that many women and infants continue to be socioeconomically vulnerable and hence in need of WIC services. These multistate findings suggest that expanded outreach to eligible nonparticipants should be considered. The information in this study can help identify the scope of WIC outreach needed and whom to target.

## Figures and Tables

**TABLE 1:** Eligibility and enrollment in the Special Supplemental Nutrition Program for Women, Infants, and Children (WIC) in 27 states and New York City — Pregnancy Risk Assessment Monitoring System (PRAMS) and California Maternal and Infant Health Assessment (MIHA), 2007–2008

State	Sample size*	Live births population†	WIC-eligible population†	WIC participants§					Eligible nonparticipants§				
	
				No.	All women		Eligible women		No.	All women		Eligible women	
			
					%	(95% CI)¶	%	(95% CI)¶		%	(95% CI)¶	%	(95% CI)¶
**Overall**	**76,049**	**4,023,136**	**2,526,026**	**1,863,195**	**46.3**	**(45.8–46.9)**	**73.8**	**(73.1–74.4)**	**662,831**	**16.5**	**(16.1–16.9)**	**26.2**	**(25.6–26.9)**
Alaska	2,764	21,528	14,998	10,386	48.2	(45.9–50.6)	69.3	(66.6–71.9)	4,612	21.4	(19.4–23.4)	30.7	(28.1–33.4)
Arkansas	3,491	75,415	56,914	42,762	56.7	(54.5–58.9)	75.1	(72.8–77.4)	14,152	18.8	(16.9–20.6)	24.9	(22.6–27.2)
California**	6,272	934,463	604,330	503,376	53.9	(52.7–55.0)	83.3	(82.1–84.5)	100,954	10.8	(10.0 –11.6)	16.7	(15.5–17.9)
Colorado	4,036	135,344	76,100	48,300	35.7	(33.5–37.8)	63.5	(60.6–66.4)	27,800	20.5	(18.8–22.3)	36.5	(33.6–39.4)
Delaware	1,893	18,611	12,074	8,607	46.2	(43.9–48.5)	71.3	(68.6–73.9)	3,467	18.6	(16.8–20.5)	28.7	(26.1–31.4)
Georgia	1,750	278,292	205,092	147,067	52.8	(49.2–56.5)	71.7	(67.8–75.6)	58,024	20.9	(17.8–23.9)	28.3	(24.4–32.2)
Hawaii	3,386	36,763	24,746	15,926	43.3	(41.7–45.0)	64.4	(62.4–66.3)	8,820	24.0	(22.5–25.4)	35.6	(33.7–37.6)
Illinois	1,706	169,046	108,018	76,584	45.3	(42.7–47.9)	70.9	(68.0–73.8)	31,435	18.6	(16.6–20.6)	29.1	(26.2–32.0)
Maryland	3,271	135,195	74,503	55,041	40.7	(38.1–43.4)	73.9	(70.6–77.1)	19,462	14.4	(12.5–16.3)	26.1	(22.9–29.4)
Maine	2,238	26,127	16,290	10,578	40.5	(38.1–42.9)	64.9	(62.0–67.9)	5,712	21.9	(19.9–23.8)	35.1	(32.1–38.0)
Michigan	1,497	119,636	69,976	52,060	43.5	(40.7–46.3)	74.4	(71.0–77.8)	17,916	15.0	(12.9–17.1)	25.6	(22.2–29.0)
Minnesota	3,068	137,628	72,107	55,689	40.5	(38.5–42.4)	77.2	(74.9–79.5)	16,418	11.9	(10.6–13.2)	22.8	(20.5–25.1)
Missouri	1,371	76,871	51,144	36,080	46.9	(43.7–50.1)	70.5	(66.8–74.3)	15,063	19.6	(16.9–22.3)	29.5	(25.7–33.2)
North Carolina	3,005	249,912	163,375	117,399	47.0	(44.8–49.1)	71.9	(69.4–74.3)	45,976	18.4	(16.7–20.1)	28.1	(25.7–30.6)
Nebraska	3,140	49,990	29,220	19,007	38.0	(36.1–40.0)	65.0	(62.4–67.7)	10,214	20.4	(18.7–22.2)	35.0	(32.3–37.6)
New Jersey	3,003	204,664	103,236	72,368	35.4	(33.8–37.0)	70.1	(67.7–72.5)	30,868	15.1	(13.7–16.5)	29.9	(27.5–32.3)
New York	2,196	229,011	125,921	92,420	40.4	(37.7–43.0)	73.4	(70.1–76.6)	33,501	14.6	(12.7–16.5)	26.6	(23.4–29.9)
Ohio	2,938	281,565	176,193	119,690	42.5	(40.1–44.9)	67.9	(65.0–70.9)	56,502	20.1	(18.1–22.1)	32.1	(29.1–35.0)
Oklahoma	4,012	103,957	77,481	59,617	57.3	(54.8–59.9)	76.9	(74.4–79.5)	17,864	17.2	(15.2–19.2)	23.1	(20.5–25.6)
Oregon	3,434	93,597	60,053	43,829	46.8	(44.2–49.4)	73.0	(70.0–76.0)	16,224	17.3	(15.3–19.4)	27.0	(24.0–30.0)
Rhode Island	2,583	22,579	13,230	10,812	47.9	(45.8–50.0)	81.7	(79.4–84.0)	2,418	10.7	(9.3–12.1)	18.3	(16.0–20.6)
South Carolina	1,450	57,711	39,916	28,770	49.9	(45.7–54.0)	72.1	(67.4–76.7)	11,146	19.3	(15.9–22.7)	27.9	(23.3–32.6)
Utah	3,520	106,320	62,764	29,842	28.1	(26.6–29.6)	47.5	(45.3–49.8)	32,922	31.0	(29.3–32.7)	52.5	(50.2–54.7)
Washington	2,958	170,591	101,467	73,829	43.3	(41.1–45.4)	72.8	(70.1–75.4)	27,638	16.2	(14.4–18.0)	27.2	(24.6–29.9)
Wisconsin	2,028	135,494	77,409	52,349	38.6	(36.4–40.9)	67.6	(64.6–70.7)	25,060	18.5	(16.5–20.5)	32.4	(29.3–35.4)
West Virginia	1,744	18,926	14,025	10,832	57.2	(53.9–60.6)	77.2	(73.9–80.6)	3,193	16.9	(14.3–19.4)	22.8	(19.4–26.1)
Wyoming	1,849	15,436	9,426	5,549	35.9	(33.5–38.4)	58.9	(55.7–62.1)	3,878	25.1	(22.9–27.3)	41.1	(37.9–44.3)
New York City	1,446	118,462	86,020	64,429	54.4	(51.1–57.7)	74.9	(71.5–78.3)	21,592	18.2	(15.6–20.8)	25.1	(21.7–28.5)

* Unweighted number of women who participated in the PRAMS and MIHA surveys.

† Population counts weighted to population of live births represented by the survey, adjusting for the sample design and nonresponse.

§ WIC participants reported that they were on WIC during pregnancy in the survey; eligible nonparticipants did not report that they were on WIC during pregnancy, but reported household incomes ≤185% of the federal poverty level in the survey or the birth certificate indicated Medicaid paid for prenatal care or delivery.

¶ Percentages and 95% confidence intervals (CIs) weighted to adjust for the sample design and nonresponse.

** California data are from MIHA; data for the other states are from PRAMS.

**TABLE 2:** Characteristics of women in 27 states and New York City delivering live-born infants — Pregnancy Risk Assessment Monitoring System (PRAMS) and California Maternal and Infant Health Assessment (MIHA), 2007–2008

Characteristic	Total			WIC participant§			Eligible nonparticipant§			Ineligible for WIC§		
			
	No.*	%†	(95% CI)†	No.*	%†	(95% CI)†	No.*	%†	(95% CI)†	No.*	%†	(95% CI)†
**Total**	**76,049**	**100**	**(100–100)**	**35,953**	**46.3**	**(45.8–46.9)**	**13,680**	**16.5**	**(16.1–16.9)**	**26,416**	**37.2**	**(36.7–37.7)**
**Race/Ethnicity**												
All non-Hispanic	61,244	75.6	(75.2–76.0)	25,566	60.8	(60.0–61.5)	11,208	79.1	(77.9–80.2)	24,470	92.6	(92.1–93.1)
White	38,464	54.2	(53.7–54.7)	12,812	35.4	(34.6–36.1)	6,962	56.9	(55.5–58.2)	18,690	76.4	(75.6–77.1)
Black	11,596	12.7	(12.3–13.0)	7,844	18.8	(18.2–19.5)	2,136	13.8	(12.8–14.8)	1,616	4.6	(4.2–4.9)
Asian/Pacific Islander	6,420	6.4	(6.1–6.6)	1,982	3.7	(3.4–4.0)	1,211	5.8	(5.2–6.4)	3,227	9.9	(9.4–10.5)
American Indian/Alaska Native	3,041	1.0	(0.9–1.0)	2,070	1.4	(1.3–1.6)	562	1.0	(0.8–1.2)	409	0.4	(0.3–0.5)
Other/Mixed	1,723	1.7	(1.5–1.9)	858	1.8	(1.5–2.0)	337	2.0	(1.5–2.4)	528	1.5	(1.2–1.7)
Hispanic	13,819	24.4	(24.0–24.8)	9,958	39.2	(38.5–40.0)	2,314	20.9	(19.8–22.1)	1,547	7.4	(6.9–7.9)
White	10,425	20.3	(19.9–20.7)	7,537	32.7	(32.0–33.4)	1,677	16.9	(15.8–18.0)	1,211	6.4	(5.9–6.8)
Black	329	0.5	(0.4–0.6)	246	0.9	(0.7–1.0)	43	0.4	(0.2–0.5)	40	0.2	(0.1–0.2)
Other	3,065	3.6	(3.4–3.8)	2,175	5.8	(5.4–6.2)	594	3.7	(3.2–4.3)	296	0.9	(0.7–1.0)
**Survey language**												
English	68,387	85.9	(85.5–86.3)	29,802	75.1	(74.4–75.8)	12,436	88.0	(87.1–88.9)	24,149	98.4	(98.2–98.6)
Spanish	7,659	14.1	(13.7–14.5)	6,151	24.9	(24.2–25.6)	1,241	12.0	(11.1–12.9)	267	1.6	(1.4–1.8)
**Education (yrs)**												
0–11	14,541	20.4	(20.0–20.8)	11,458	35.3	(34.6–36.0)	2,664	20.8	(19.6–22.0)	419	1.7	(1.5–2.0)
12	21,628	28.0	(27.5–28.5)	13,732	38.3	(37.5–39.1)	4,626	34.8	(33.5–36.2)	3,270	12.2	(11.6–12.8)
≥13	38,718	51.7	(51.1–52.2)	10,133	26.4	(25.7–27.1)	6,164	44.3	(42.9–45.7)	22,421	86.1	(85.5–86.7)
**Age group (yrs)**												
<18	2,537	3.1	(2.8–3.3)	2,077	5.5	(5.1–5.9)	415	2.7	(2.3–3.2)	45	0.1	(0.1–0.1)
18–24	23,697	29.8	(29.2–30.3)	16,655	45.5	(44.6–46.3)	4,900	35.6	(34.3–37.0)	2,142	7.6	(7.1–8.1)
25–39	47,510	64.4	(63.9–65.0)	16,578	47.5	(46.6–48.3)	7,965	58.9	(57.5–60.3)	22,967	87.9	(87.3–88.5)
≥40	2,302	2.8	(2.6–2.9)	642	1.5	(1.3–1.7)	398	2.7	(2.3–3.1)	1,262	4.3	(4.0–4.7)
**Total live births**												
1st live birth	31,888	41.2	(40.6–41.7)	14,852	40.2	(39.4–41.1)	5,132	37.2	(35.8–38.6)	11,904	44.1	(43.2–45.0)
2nd–3rd birth	35,209	48.3	(47.7–48.8)	15,912	46.3	(45.4–47.1)	6,346	47.7	(46.3–49.1)	12,951	50.9	(50.0–51.8)
4th birth or greater	8,615	10.6	(10.2–10.9)	5,025	13.5	(12.9–14.1)	2,121	15.1	(14.1–16.1)	1,469	5.0	(4.6–5.3)
**Not married at delivery**	29,988	38.7	(38.1–39.2)	22,225	62.3	(61.5–63.1)	5,911	43.8	(42.4–45.2)	1,852	7.1	(6.6–7.6)
**Income as % of FPL** ¶												
0–100% FPL	26,473	32.2	(31.6–32.7)	20,852	55.5	(54.7–56.4)	5,621	39.1	(37.7–40.5)	0	0.0	—
101%–185% FPL	14,584	18.6	(18.1–19.0)	8,313	23.6	(22.9–24.4)	6,271	46.2	(44.8–47.6)	0	0.0	—
≥185% FPL	29,780	41.8	(41.3–42.4)	2,563	7.6	(7.2–8.1)	801	6.7	(6.0–7.4)	26,416	100.0	—
Missing	5,212	7.4	(7.1–7.8)	4,225	13.2	(12.6–13.8)	987	8.0	(7.1–8.9)	0	0.0	—
**Preconception health coverage**												
Medicaid	12,957	17.7	(17.3–18.1)	10,423	31.0	(30.2–31.8)	2,302	17.4	(16.4–18.5)	232	1.4	(1.1–1.6)
Private/Other	40,098	53.0	(52.5–53.6)	8,930	23.2	(22.5–23.9)	6,051	42.5	(41.1–43.9)	25,117	94.6	(94.2–95.0)
Uninsured	22,630	29.3	(28.7–29.8)	16,340	45.8	(44.9–46.6)	5,267	40.1	(38.7–41.5)	1,023	4.0	(3.6–4.4)
**Prenatal health-care coverage**												
Medicaid/Medi-Cal	32,244	43.3	(42.7–43.8)	25,804	75.9	(75.1–76.6)	6,440	51.6	(50.2–53.1)	0	0.0	
Private/Other	36,545	53.0	(52.5–53.6)	6,357	20.1	(19.5–20.8)	5,161	40.8	(39.4–42.2)	25,027	98.2	(98.0–98.4)
Uninsured	2,906	3.7	(3.5–4.0)	1,429	4.0	(3.6–4.4)	907	7.6	(6.7–8.4)	570	1.8	(1.6–2.0)
**Prenatal care initiation**												
No prenatal care	1,016	1.8	(1.7–2.0)	524	2.3	(2.0–2.6)	358	2.7	(2.2–3.1)	134	0.9	(0.7–1.0)
1st trimester	58,684	82.3	(81.9–82.8)	25,519	76.0	(75.3–76.7)	9,639	75.2	(73.9–76.4)	23,526	93.1	(92.6–93.6)
2nd trimester	10,658	13.6	(13.3–14.0)	6,764	18.7	(18.0–19.4)	2,346	18.4	(17.3–19.5)	1,548	5.4	(5.0–5.8)
3rd trimester	1,812	2.2	(2.1–2.4)	1,111	3.0	(2.7–3.3)	524	3.8	(3.3–4.3)	177	0.6	(0.5–0.8)
**Unintended pregnancy**	31,752	42.4	(41.9–43.0)	19,300	55.8	(55.0–56.7)	6,738	51.1	(49.6–52.5)	5,714	22.1	(21.3 –22.9)
**Prepregnancy BMI** **												
Underweight (<18.5)	3,568	4.1	(3.8–4.3)	1,872	4.4	(4.0–4.7)	792	5.1	(4.5–5.7)	904	3.2	(2.9 –3.6)
Normal (18.5–24.9)	36,141	48.1	(47.6–48.7)	14,695	40.6	(39.7–41.4)	6,507	47.4	(45.9–48.8)	14,939	57.9	(57.0–58.8)
Overweight (25.0–29.9)	17,094	23.2	(22.7–23.7)	8,027	23.5	(22.8–24.2)	3,075	23.0	(21.8–24.2)	5,992	23.0	(22.2–23.7)
Obese (≥30)	14,662	18.0	(17.6–18.5)	8,009	21.0	(20.3–21.7)	2,499	17.8	(16.8–18.9)	4,154	14.4	(13.7–15.0)
Missing	4,584	6.6	(6.3–6.8)	3,350	10.6	(10.0–11.1)	807	6.7	(5.9–7.4)	427	1.5	(1.3–1.7)
**Preconception smoker**	17,207	21.2	(20.7–21.7)	10,612	27.5	(26.7–28.3)	3,614	25.1	(23.9–26.4)	2,981	11.8	(11.2–12.4)
**Prior LBW or preterm birth** ††												
No previous live birth	31,888	42.7	(42.1–43.3)	14,852	41.7	(40.8–42.6)	5,132	38.7	(37.3–40.1)	11,904	45.7	(44.8–46.6)
No LBW or preterm birth	31,534	48.1	(47.5–48.6)	14,409	47.3	(46.4–48.1)	6,035	50.7	(49.3–52.1)	11,090	47.9	(47.0–48.8)
LBW and/or preterm birth	8,651	9.2	(8.9–9.5)	4,844	11.1	(10.5–11.6)	1,814	10.6	(9.7–11.4)	1,993	6.4	(5.9–6.8)
**Markers of risk** §§												
One or more	61,344	79.1	(78.7–79.5)	34,970	97.1	(96.8–97.4)	12,420	90.7	(90.0–91.5)	13,954	51.5	(50.6–52.4)
Two or more	48,016	61.2	(60.7–61.7)	32,470	90.3	(89.8–90.8)	10,277	75.1	(73.9–76.3)	5,269	18.9	(18.2–19.6)
Three or more	36,917	46.7	(46.1–47.2)	27,653	76.2	(75.5–76.9)	7,779	56.9	(55.5–58.3)	1,485	5.4	(5.0–5.8)
Four or more	25,404	31.2	(30.7–31.8)	20,044	54.0	(53.2–54.9)	5,073	36.0	(34.6–37.4)	287	0.8	(0.6–1.0)

**Abbreviations:** CI = confidence interval; WIC = Special Supplemental Nutrition Program for Women, Infants, and Children; BMI = body mass index; LBW = low birth weight; FPL = federal poverty level.

* Unweighted number of women who participated in the PRAMS and MIHA surveys.

† Percentages and 95% CIs weighted to adjust for sample design and nonresponse.

§ WIC participants reported that they were on WIC during pregnancy in the survey; eligible nonparticipants did not report that they were on WIC during pregnancy, but reported household incomes ≤185% of the FPL in the survey or the birth certificate indicated Medicaid paid for prenatal care or delivery; nonparticipants in WIC or Medicaid with incomes >185% FPL were considered ineligible for WIC.

¶ Incomes ≤185% FPL are WIC-eligible.

** BMI calculated as (weight [kg]/height [m]2) where values 0–18.49 = underweight, 18.5–24.9 = healthy weight, 25–29.9 = overweight, and ≥30 = obese.

†† Low birth weight = less than 5 pounds, 8 ounces (<2,500 g); preterm birth is before 37 weeks gestation.

§§ Markers of risk include either age <18 years or <12 years of education (composite variable); 4th live birth or greater; not married; poor; Medicaid or uninsured before pregnancy; unintended pregnancy; underweight or obese before pregnancy; prenatal smoking; and any history of prior poor birth outcome.

## Footnotes

* FPL for a family of four was $20,650 in 2007 and $21,200 in 2008, and 185% of FPL was $38,203 in 2007 and $39,220 in 2008. Additional information on WIC eligibility requirements is available at http://www.fns.usda.gov/wic. Additional information on the FPL is available at http://aspe.hhs.gov/poverty/figures-fed-reg.cfm.
